# Mechanics guides the design of high-performance switchable adhesives

**DOI:** 10.1093/nsr/nwae240

**Published:** 2024-07-13

**Authors:** Kevin T Turner

**Affiliations:** Department of Mechanical Engineering and Applied Mechanics, University of Pennsylvania, USA

In recent years there has been a significant push to develop switchable adhesives that have high adhesion in one state and low adhesion in another. Switchable adhesives hold promise for transforming the design of robotic end-effectors, enhancing part handling in manufacturing, and improving the mounting of wearables. Due to their reversible nature, switchable adhesives typically rely on surface forces (e.g. van der Waals interactions). Tuning surface forces on-demand is challenging, and thus switchable adhesives often exploit innovative mechanical designs, such as mushroom-shaped [1] or composite fibrils [2]. These designs enhance stress uniformity at the fibril-surface interface to achieve high adhesion strength and allow the adhesion to be reduced by modifying the loading on the fibrils (e.g., application of shear or fibril buckling) or by altering the mechanics using a tunable stiffness material [3,4].

Individual fibrils and fibril arrays are common in switchable adhesives; thus, the mechanics of fibril contacts play a crucial role in adhesive performance. If a fibril is smaller than a critical radius, the contact behaves in a strength-limited or DMT-like manner, and the intrinsic adhesion strength of the interface is realized [5]. Conversely, when fibrils are larger than this critical radius, failure occurs via the propagation of an interface crack from the fibril's edge, a JKR-like adhesion, and the strength is lower. The critical fibril radius depends on the elastic modulus and the adhesive interactions. For compliant materials, which adhere easily due to their ability to conform to roughness, the critical fibril size is small, and achieving DMT-like behavior is challenging [5]. The critical fibril size is larger for stiffer materials, but these materials cannot usually accommodate roughness to allow for adhesion.

**Figure 1.**
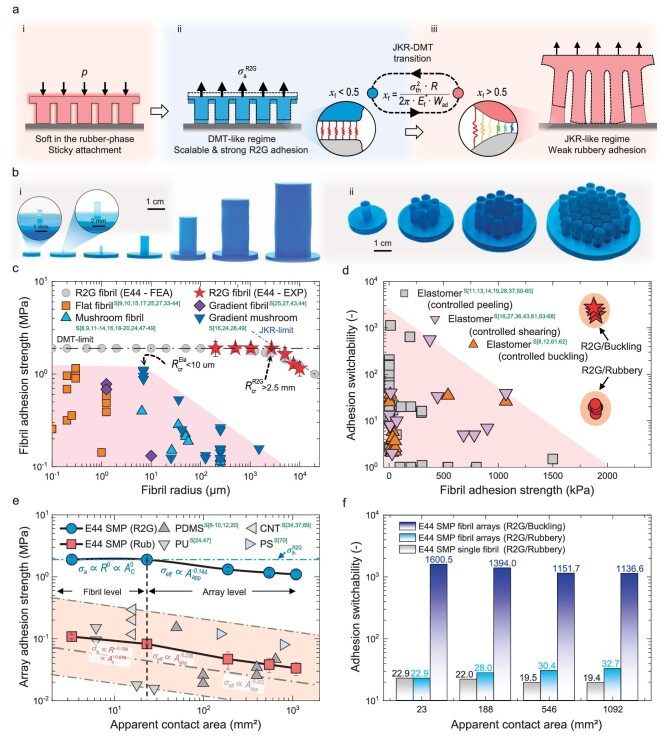
R2G fibrillar adhesives that use an R2G phase change in SMPs to transition between the JKR and DMT regime to achieve high strength and highly switchable adhesion. (a) Schematic of the attachment and detachment process. (b) Photos of (i) the SMP adhesive fibrils and (ii) fibril arrays. (c and d) Experimental results showing the (c) adhesion strength and (d) adhesion switchability of the R2G fibrils compared to elastomer fibrils. (e and f) Experimental results of the (e) adhesion strength and (f) switchability of the R2G arrays compared to elastomer fibril arrays. Reprinted from Ref. [6]. Citations in Fig. 1 should be referred to Ref. [6].

Linghu *et al*. [6] recently overcame this limitation by using a shape-memory polymer (SMP) that is rubbery with a low modulus at elevated temperatures and glassy with a high modulus at room temperature. Their SMP fibrillar adhesive is contacted in the heated rubbery state, achieves high DMT-like adhesion when cooled and glassy, and switches to a lower adhesion JKR-like contact when heated (Fig. 1a and b). By leveraging the JKR-DMT transition, the team realized switchable adhesives with high strength (∼2 MPa) and excellent switchability (∼2000×) in mm-scale fibrils (Fig. 1c and d). Moreover, high adhesion strength and tunability were also achieved in fibril arrays (Fig. 1e and f). The impressive performance reported in this study stems from a mechanics-based design strategy used to select an optimal fibril radius that enables DMT-like behavior with high adhesion in the SMP's glassy state and JKR-like adhesion in the rubbery state. These highly effective switchable adhesives with mm-scale dimensions are significantly easier to manufacture and deploy than the more commonly studied microscale fibrils. The team demonstrated the versatility of their SMP adhesives across various applications, notably showing the ability to hold and detach objects ranging from 0.3 to 60 kg using arrays [6]. Given the remarkable performance, these adhesives are likely to have a near-term impact in the field of robotics for grasping and climbing robots, and in pick-and-place manufacturing processes.
